# Supplementary material for: Investigating microbial population structure and function in the chicken caeca and large intestine over time using metagenomics
Source: BMC Res Notes. 2025 Aug 15;18:355. doi: 10.1186/s13104-025-07441-7 (PMC12357378; doi:10.1186/s13104-025-07441-7)
Supplement: Supplementary file 2 — Recovered nutrient cycles including Carbon, Nitrogen, and Sulphur cycles for all the metagenomic assembled genomes given as PDF images, and identifiable through bin numbers. [file 13104_2025_7441_MOESM2_ESM.zip › Supplementary Data2/draw_nitrogen_cycle_total.pdf]

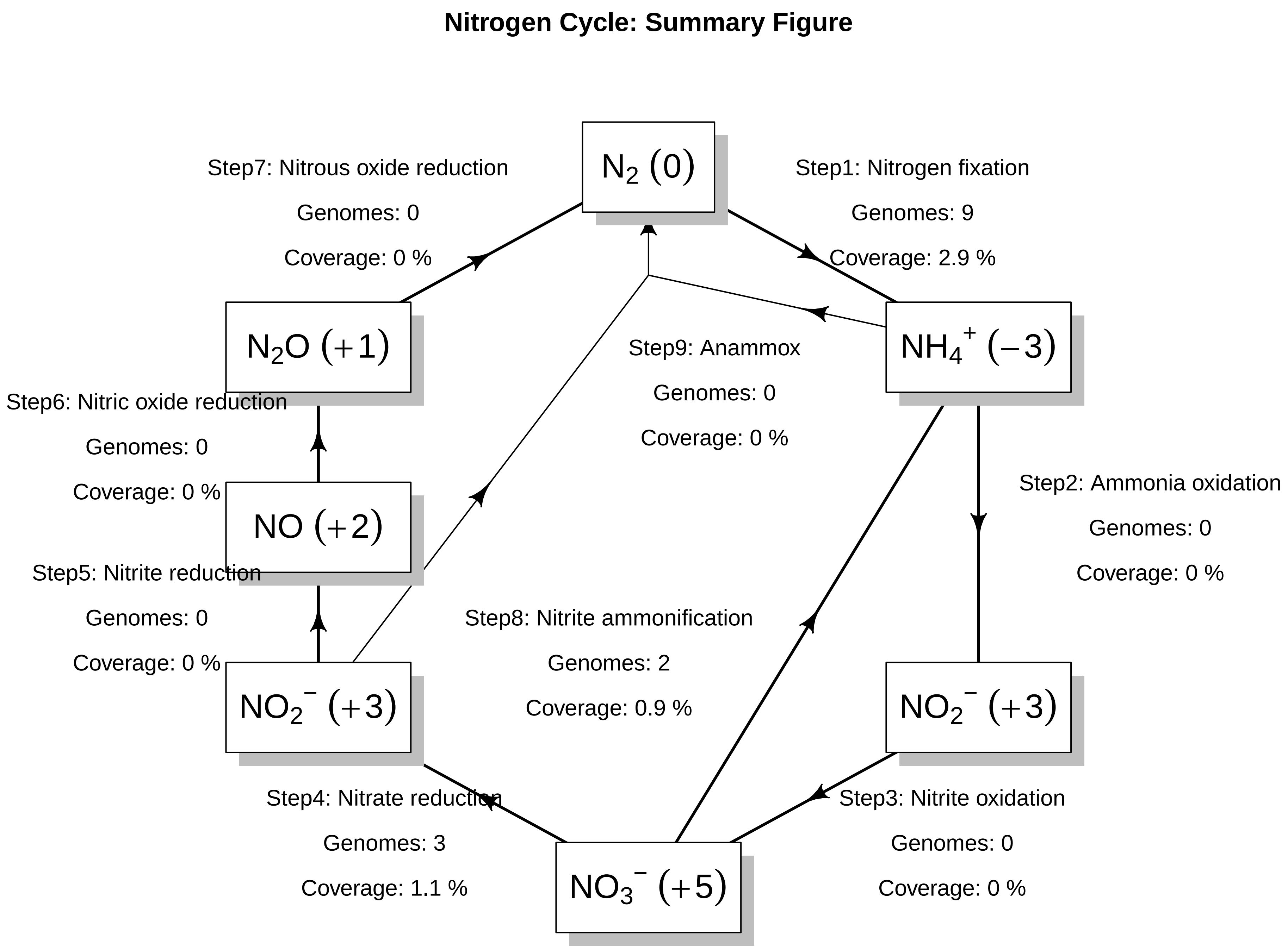

Nitrogen Cycle: Summary Figure
N_2 (0)
Step1: Nitrogen fixation
Genomes: 9
Coverage: 2.9 %
NH_4^+ (−3)
Step2: Ammonia oxidation
Genomes: 0
Coverage: 0 %
NO_2^- (+3)
Step3: Nitrite oxidation
Genomes: 0
Coverage: 0 %
NO_3^- (+5)
Step4: Nitrate reduction
Genomes: 3
Coverage: 1.1 %
NO_2^- (+3)
Step5: Nitrite reduction
Genomes: 0
Coverage: 0 %
NO (+2)
Step6: Nitric oxide reduction
Genomes: 0
Coverage: 0 %
N_2O (+1)
Step7: Nitrous oxide reduction
Genomes: 0
Coverage: 0 %
Step8: Nitrite ammonification
Genomes: 2
Coverage: 0.9 %
Step9: Anammox
Genomes: 0
Coverage: 0 %
